# Menthol in Livestock: Unveiling Its Multifaceted Properties and Future Potential for Sustainable Agriculture

**DOI:** 10.3390/ijms26062679

**Published:** 2025-03-17

**Authors:** Brandon Bernard, Himani Joshi, Peixin Fan

**Affiliations:** 1 Department of Biochemistry, Nutrition & Health Promotion, College of Agriculture and Life Sciences, Mississippi State University, Starkville, MS 39762, USA; bb2842@msstate.edu; 2 Department of Animal and Dairy Sciences, College of Agriculture and Life Sciences, Mississippi State University, Starkville, MS 39762, USA; hj532@msstate.edu; 3 Institute for Genomics, Biocomputing & Biotechnology, Mississippi State University, Starkville, MS 39762, USA

**Keywords:** menthol, peppermint, livestock, metabolism, microbiota, transient receptor potential channels

## Abstract

Menthol, the primary active compound in the widely cultivated peppermint plant (*Mentha piperita*), is well known for its use in human products such as topical analgesics and cold remedies. Menthol’s cooling sensation and ability to locally modulate pain through interactions with transient receptor potential channels make it a valuable bioactive compound. In recent years, menthol’s antimicrobial, anti-inflammatory, and antioxidative properties have drawn attention in the livestock industry as a natural alternative to synthetic antibiotics in feed additives. This review comprehensively examines the existing literature to assess menthol’s effects on animal growth performance, product quality, immune function, gastrointestinal microbial ecosystems, and metabolism across various livestock species. Notably, menthol shows potential for improving feed efficiency, mitigating chronic inflammation and oxidative stress, inhibiting environmental and gastrointestinal pathogens, and enhancing calcium absorption. However, optimal dosages, treatment durations, synergies with other phytogenic compounds, and regulatory mechanisms require further investigation. Additionally, with increasing global temperatures and growing concerns about animal welfare, menthol’s cooling, methane-reducing, and analgesic properties present promising opportunities for advancing sustainable livestock practices.

## 1. Introduction

Menthol is a widely used phytogenic (plant-derived) compound and the principal component of various mint species, including *Mentha arvensis*, *Mentha canadensis*, *Mentha aquatica*, and *Mentha piperita* [1,2]. *Mentha piperita* (*M. piperita*), commonly known as peppermint, is an especially popular hybrid species cultivated worldwide for use in a variety of consumer products [2]. The popularity of *M. piperita* largely stems from its high levels of menthol relative to other species. In addition to natural sources in Mentha species, menthol can also be synthesized chemically at a cost-effective rate (USD 0.2/g for chemical grade; USD 0.1/g for food grade). Worldwide production of menthol is approximately 34,000 metric tons per year, with synthetic menthol accounting for about 60% of this total [3]. Most famously, menthol is capable of exerting potent cooling and analgesic sensations, which are utilized in products such as muscle cooling gels for athletes and cough drops. It exerts potent antimicrobial activity [4,5,6,7], as well as possessing anti-inflammatory and antioxidant properties [5,8,9,10,11,12]. Controversially, these properties are exploited in menthol cigarettes, where menthol reduces irritation caused by smoke inhalation [13]. Furthermore, menthol and peppermint have been found to influence appetite and salivation, leading to their common use in gum and candies [14,15,16,17].

**Figure 1:**
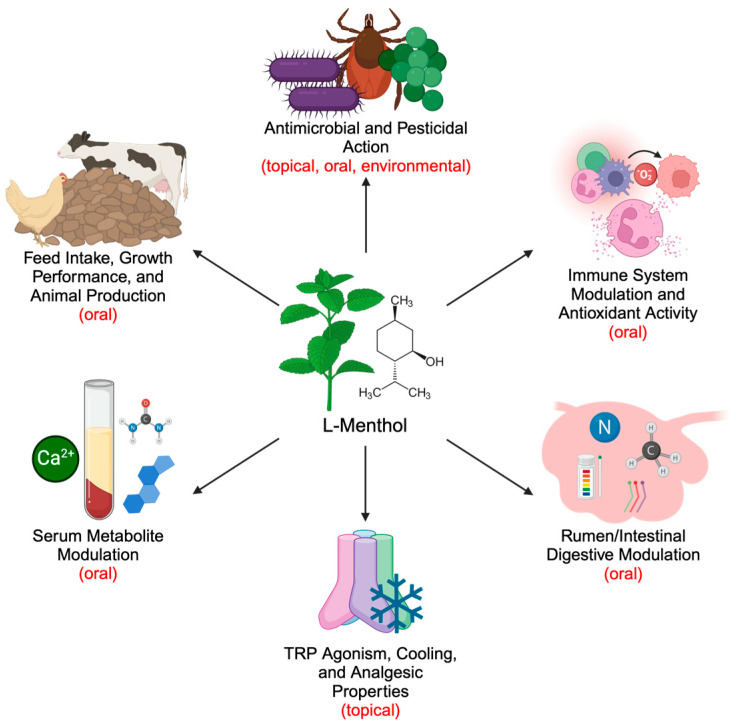
Effects of menthol and menthol-containing compounds such as peppermint oil in humans, livestock, and other animals. Effects of oral supplementation in livestock include regulation of feeding behaviors, increased feed efficiency, and improved milk, meat, and egg production. Oral and topical administration regulates levels of immune cells, immunoglobulins, pro-inflammatory cytokines, and reactive oxygen species, as well as facilitating antibacterial and pesticidal action. Antibacterial action results in modified digestive performance and production of microbial metabolites, such as methane and short-chain fatty acids, in the gastrointestinal tract. Changes in feeding and digestion subsequently affect concentrations of serum parameters, such as glucose, cholesterol, fatty acids, and urea. Additionally, transient receptor potential (TRP) channel agonism increases absorption of cations such as Ca^2+^ and ammonium. In human and mouse models, TRP channel agonism produces cooling and analgesic sensations. This figure was created using BioRender.

While menthol has traditionally played a role in a wide array of human-directed applications, it has also received attention for use in livestock such as poultry and ruminants in recent years. Menthol holds several potential uses for increasing the efficiency of livestock production as well as improving animal welfare. Its appetite-modulating properties could contribute to improving animal growth performance and product yields. Menthol also possesses antimicrobial effects that may present a viable alternative to traditional antibiotics, which face increasing regulatory restrictions in livestock production due to rising antimicrobial resistance [18,19]. Through this antimicrobial action, menthol can additionally regulate nutrient digestion and methane emissions by influencing gastrointestinal microbiota [20,21]. Given that ruminants heavily rely on their gastrointestinal microbiota for digestive function, menthol-induced alterations in these microbes could alter the entire metabolic profile of the animal. Furthermore, when combined with other interventions, menthol’s cooling properties hold the potential for mitigating heat stress, a challenge increasingly facing livestock production [22,23]. In recent years, the study of phytogenic feed additives in livestock production has become widespread [24,25,26]. However, despite the well-documented biochemical properties of menthol, research on peppermint and other menthol-containing products in livestock contexts remains limited, with studies utilizing pure menthol being even more scarce. This review systematically summarizes current literature that investigates the effects of menthol and other menthol-rich compounds in livestock and provides insight for future directions of study (Figure 1).

## 2. Physical, Chemical, and Biological Properties of Menthol

Menthol is classified as a monocyclic monoterpene, a molecule derived from two isoprene units [27]. Menthol forms a characteristic solid, white, crystalline form at room temperature and is sparingly water soluble [1]. There are four optical isomers of menthol; however, L-menthol is the most common form found in nature and the most studied for its biological effects. The Food and Drug Administration (FDA) classifies menthol as Generally Recognized as Safe (GRAS) [28]. When ingested, menthol is readily absorbed in the intestines, where it is quickly transported to the liver, glucuronidated to menthol glucuronide, and largely excreted in urine [29]. Several metabolites of menthol have been identified beyond menthol glucuronide, including oxidated, hydroxylated, and sulfated products. Prominent menthol metabolites found in urine include 3,8-dihydroxy-p-menthane-7-carboxylic acid and p-Menthane-3,8-diol. These metabolites are largely considered inert and nontoxic, though most are understudied [29,30]. However, before absorption and metabolism, menthol holds the potential to exert gastroprotective effects [8]. Menthol’s antimicrobial activity is partly attributed to its ability to disrupt the lipid membrane of microbial cells [31]. Additionally, menthol’s hydroxyl groups allow for reactive oxygen species scavenging and antioxidant activity [8,12,32].

**Figure 2:**
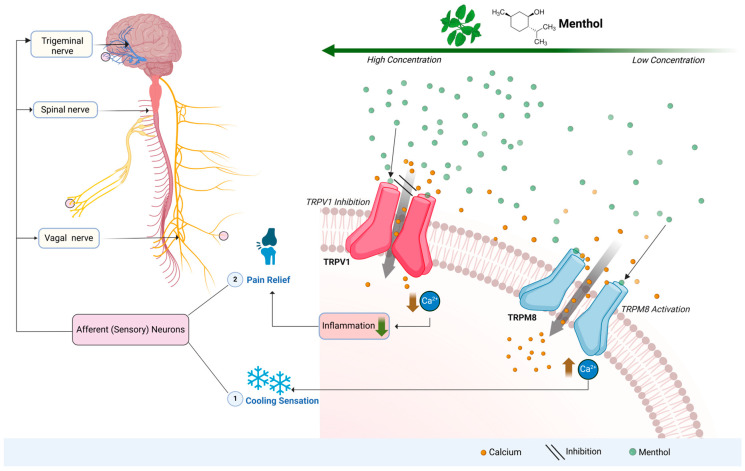
Menthol induces cooling and pain relief by modulating TRP ion channels. At concentrations of 60–120 μM in vivo, menthol binds to and activates TRPM8, a cold-sensitive ion channel expressed on sensory neurons of the vagal, spinal, and trigeminal nerves. This triggers calcium influx, generating neuronal signals perceived as cooling (<15 °C). At higher concentrations (>10 mM in vitro, 5 mM in vivo), menthol inhibits TRPV1, reducing Ca^2+^ influx and suppressing inflammatory responses and thereby modulating pain perception, leading to pain relief. This figure was created using BioRender.

Among menthol’s most renowned biological effects is its ability to induce a cooling and pain-relieving sensation, achieved through the activation of transient receptor potential (TRP) cation channels, primarily TRP melastatin 8 (TRPM8), a Ca^2+^ channel [33]. TRPM8 is expressed primarily on the afferent (sensory) neurons of the vagus nerve, the spinal nerve, and the trigeminal nerve [34,35,36], which are integral pathways for transmitting sensory signals to the central nervous system. Menthol, particularly at concentrations of 60–120 μM in vivo, binds to and activates TRPM8, triggering a cold sensation typically perceived at temperatures below 15 °C [37,38]. The activation of TRPM8 leads to calcium influx, which generates neuronal signals perceived as cold by the brain and spinal cord [37,39,40]. Another TRP channel, TRP vanilloid 1 (TRPV1), is activated by temperatures above 43 °C and plays a key role in detecting noxious heat, which can trigger the burning pain sensation, and is also involved in inflammatory responses [37,38]. Menthol in higher concentrations of 5 mM in vivo and 10 mM in vitro can directly inhibit the activity of TRPV1 channels by eliciting inhibition to the inflammatory response due to reduced Ca^2+^ influx [41]. This modulates the level of pain perception, leading to pain relief [37,38,41,42,43] (Figure 2). TRPA1, which detects extreme cold (<8 °C) and irritants, is also co-activated through activation of TRPV1 channels, leading to cold hypersensitivity [40,44,45]. High concentrations of menthol (0.3%) can also activate the TRPA1 receptor, leading to greater irritation and nociception [40,41,44,45]. Interestingly, TRPM8, TRPV1, and TRPA1 are co-expressed in certain afferent neurons, enabling the sensation of both cold and hot stimuli, which explains phenomena like paradoxical cold-induced burning pain [33,39,40,45].

## 3. Impact of Menthol and Menthol-Rich Supplements on Feed Intake, Growth Performance, and Animal Production

In the livestock industry, maintaining feed intake and optimizing product yield (meat, milk, eggs) are key priorities. While research on menthol and peppermint’s effects is substantial, most studies focus on menthol-containing compounds or peppermint as feed additives, with limited investigation into pure menthol as a supplement (Table 1).

### 3.1. Feed Intake and Feeding Behavior

The effect of menthol or menthol-containing feed additives on feed intake has been investigated in cattle. In lactating cattle, supplementation with 3 g/day of the menthol-containing blend Aromix^®^ (Masa Egypt Company, Alexandria, Egypt) (133 mg/g) increased lactating cattle’s feed intake from 13.4 to 13.8 kg/day, but a higher dose of 6 g/day reduced the intake to 13.0 kg/day [48]. Other cattle studies also reported increased feed intake after supplementation with peppermint [54] or the peppermint-containing supplement BioHerbal^®^ (Pars-Imen-Daru Herbal Medicines Development Co., Tehran, Iran) [49]. In sheep, feed intake improved after supplementation with peppermint [57] and OAX17 (PerformaNat GmbH, Berlin, Germany), a commercial blend comprising about 90% menthol [55,56]. However, several studies reported no significant changes in feed intake after menthol or menthol-containing compound supplementation, including nonlactating Holstein cattle fed with 6.7–67 mg per kg of pure menthol [46], lactating Friesian and Holstein cattle supplemented with 5% peppermint or 1.2 to 6 g/day of menthol-containing commercial supplements [50,51,52], and male Murrah buffalos supplemented with 2.5% garlic bulb and peppermint over 21 day periods [58].

In poultry, feed intake increased after newborn Ross 308 chickens were supplemented with 26–78 mg/kg of pure menthol during two separate 35 day trial periods [59]. Significant increases in feed intake after mint feeding were observed in a variety of life stages, from 1-day-old chicks to 64-week-old laying hens [61,65,66,78,79]. In contrast, no changes to feed intake were found in most other studies, which supplemented chickens of various life stages with peppermint [62,67,68,69,70,71,72,73] or 100–150 mg of a phytogenic blend of menthol, anethol, and eugenol per kg [63]. Narimani et al. even reported a decrease in average feed intake from 160.19 g/d to 147.6 g/d in young chickens fed a blend of 1% peppermint, 0.5% oregano, and 0.5% ziziphora for 42 days [64]. A different study similarly observed a decrease in feed intake in young chickens at 15 g/kg of peppermint supplementation, but not at lower concentrations [74]. These inconsistencies suggest that menthol’s effects are dosage-dependent and may be influenced by mixed phytogenic compounds.

Menthol compounds also influence feeding behavior in ruminants. After 6.7–67 mg/kg menthol supplementation, the average feeding time of cattle tended to increase from 39.8 min to 47.2 min [46], and short-term increases in salivation rate were detected [47]. Similar results persist with impure supplements. In a 4-week study using Suffolk sheep, a linear increase in feeding time and frequency was reported after supplementation with 80–160 g/day of OAX17 [55]. Lactating Holstein cattle had an increased feeding time after being supplemented with peppermint at 5% of their diet for 22 days [52]. The potential mechanism of menthol effects on increasing feeding time could be attributed to its reduction in the palatability of the feed and stimulation of TRP channels, with TRPM8 potentially modulating feed intake [52,55].

### 3.2. Growth Performance and Animal Production

As with increased feed intake, improvement of body weight in young chickens fed menthol was reported [59], and some studies also observed improved weight gain after supplementation with menthol-containing compounds [61,66,75]. However, several other studies found no significant changes to the body weight of sheep [56] or chickens [62,64,70,76,80]. Additionally, Gurbuz and Ismael found that the average chicken body weight decreased over a 35-day trial, with an average of 2413 g for the control group compared to 2178 g for birds supplemented with 15 g of peppermint per kg of feed [74]. This corresponds with their observed decrease in feed intake after peppermint supplementation. 

While the effects of menthol on feed intake varied, and few studies observed changes in body weight, evidence for improvements in the feed conversion ratio (FCR) is far more consistent. Pure menthol supplementation at 26–78 mg/kg of feed during the first 21 days of life improved the average chicken FCR [59]. Many other poultry studies at varying life stages also observed FCR improvements after supplementation with peppermint [65,66,68,72,75,76,77,78,80] or a combination of compounds including peppermint, menthol, anethol, oregano, etc. [61,63,64]. However, several studies on young chickens reported no difference in chicken FCRs [62,70,71,74]. Khodambashi Emami et al. observed improvements in the FCR of chickens supplemented with 200 mg of peppermint/kg of feed but not when increased to 400 mg/kg, further suggesting the dose-dependent effectiveness of menthol on feed efficiency and growth performance [69]. However, sheep supplemented with 3% peppermint for 90 days showed no FCR improvement [57]. The mechanisms behind improvements in FCRs are not entirely clear, but several studies suggest the antimicrobial and antioxidant qualities of menthol help to stabilize the gut and improve digestion, which will be discussed in detail in the following sections [59,61,71].

Menthol supplementation also led to improvements in meat, milk, and egg production. In chickens, pure menthol supplementation linearly decreased meat abdominal fat and cook loss percentage [59]. Other menthol-containing additives also reduced abdominal fat, increased carcass yield, and enhanced overall production [64,68,70]. Mehri et al. found a decrease in carcass drip loss and malondialdehyde (MDA), which contributes to meat odor and spoilage, in Japanese quail supplemented for 5 weeks with peppermint at 10–40 g/kg feed [81]. These meat-quality improvements are attributed to peppermint’s antioxidant activity, which will also be discussed in detail. Other studies found no significant changes in the carcass characteristics of young chickens supplemented with peppermint [67,74,75]. Furthermore, feeding 1 week old chickens 0.2% peppermint (~70 mg menthol/kg feed) for 42 days resulted in an increase in abdominal fat [67]. The researchers attributed this to growth promotion due to the supplement, as fast growth is linked to increased fat. Chicken egg quality followed a similarly positive trend, with improvements in laying rate, cleanliness, weight, and shell thickness found after laying hens were supplemented with peppermint [60,65,73,77,78,79]. Rahman et al. reported minimal improvements to egg quality after peppermint supplementation up to 200 mg/kg, but improvements in egg yolk color were recorded on day 28 of a 56-day trial period [72].

Improvements in both yield and quality of milk were found after supplementing lactating cows with various commercial products containing menthol, including BTX12 (PerformaNat GmbH, Berlin, Germany) (>80% menthol), Aromix^®^, and BioHerbal^®^ [48,49,50,51]. These trials ranged from 20 days to 3 months. In contrast, other studies did not observe changes in milk production after 5% peppermint supplementation for 22 days [52,53]. These results suggest that effects on milk production may only be observed over long-term supplementation periods. Additionally, commercially formulated supplement blends may be more effective for improving milk performance than peppermint alone. However, some studies reported diminished milk output at higher supplementary concentrations, suggesting menthol’s antimicrobial properties may negatively affect digestion and output if not properly dosed [48,51]. There exists evidence suggesting cattle supplemented with herbs, such as oregano and caraway, may positively affect the aroma of produced milk [82]. Menthol and peppermint may confer similar attributes to the milk of supplemented dairy cows, which needs to be further explored.

These results provide strong evidence for menthol’s potential to improve livestock production. However, future research is needed to better elucidate the specific mechanisms behind these effects and determine optimal feed supplementation so as to maximize beneficial biochemical effects and minimize detrimental ones.

## 4. Anti-Inflammation, Immune System Modulation, and Anti-Oxidation Properties of Menthol

### 4.1. Anti-Inflammation

Inflammation is a crucial component of the immune response, facilitating the removal of harmful stimuli and the repair of damaged tissue. It can be triggered by various stressors, including infection, heat stress, and dietary factors, which stimulate the release of pro-inflammatory cytokines, such as interleukin-6 (IL-6), IL-1β, and tumor necrosis factor-α (TNF-α) [83,84]. While inflammation is essential for immune defense and tissue recovery, excessive production of pro-inflammatory cytokines and leukocytes in response to prolonged stress can lead to chronic inflammation. In livestock, chronic inflammation has been associated with several adverse effects, including impaired growth performance and metabolic function, increased mortality, intestinal barrier dysfunction (leaky gut), reduced egg production in poultry, and decreased milk yield in dairy cows [84,85,86]. Menthol’s anti-inflammatory properties have been extensively studied in vivo and in vitro [1,12], indicating the potential of peppermint and other menthol products to improve livestock health through the reduction of excess inflammation.

In vitro, pure menthol’s ability to alleviate mastitis induced by lipopolysaccharide (LPS) challenge in bovine mammary gland epithelial cells (BMECs) was investigated [87]. Pre-treatment with 10 µM of menthol mitigated the negative effects of LPS on milk lipid and protein production in BMECs while also reducing the abundance of pro-inflammatory cytokines, including IL-6, IL-1β, and TNF-α. This effect was attributed to menthol-induced activation of the unc-51 like kinase 1 (ULK1) and nuclear factor-E2 associated factor 2 (Nrf-2) pathways, which initiate and sustain autophagy of proinflammatory cytokines and enzymes, such as cyclooxygenase-2 and inducible nitric oxide synthetase, both of which are involved in the inflammatory response [87]. Similarly, in vivo reductions in pro-inflammatory IL-18 in chickens and the inflammation marker serum amyloid A in cattle were observed after supplementation with 100–150 mg/kg of a menthol-containing blend and 0.04% Digestarom^®^ (DSM GmbH, Grenzach-Whylen, Germany) (menthol, thymol, and eugenol), respectively [63,88]. In contrast, increases in pro-inflammatory IL-6 and TNF-α were observed in the serum of chickens supplemented with a *Mentha arvensis*/*Geranium thunbergii* mixture at a concentration of 0.01% of feed [79]. Overall, menthol appears to possess modulatory capabilities for the inflammatory response through reductions in pro-inflammatory mediators.

### 4.2. Immune System Modulation

There is also evidence that menthol influences leukocyte concentrations. In sheep, white blood cell counts significantly increased following supplementation with 30 mL of peppermint oil per kg of feed [89]. Neutrophils are significant producers of reactive oxygen species (ROS) in the immune system and serve as biomarkers for inflammation [10,90,91]. Avians such as poultry lack neutrophils but possess functionally similar heterophils [92,93]. The heterophil to lymphocyte (H/L) ratio has been demonstrated as a marker of avian health, with a lower H/L ratio indicating reduced stress and superior immune response and survival [94,95,96,97]. Thus, reducing excess neutrophil and heterophil activity is desirable for animal health. Healthy growing Suffolk sheep exhibited a significant decrease in serum neutrophils supplemented with 160 mg of OAX17 per day over a 28 day trial [56]. The average H/L ratios of healthy Ross 308 chickens supplemented with 200 ppm of peppermint per kg of body weight in the forms of alcoholic extract, encapsulated nanoparticles, and encapsulated microparticles all decreased [76]. Similarly, decreased H/L ratios in young Hubbard chickens supplemented with peppermint at 0.25–1.5% of feed for 6 weeks were observed [66]. In contrast, Fallah et al. reported a significant increase in heterophil concentrations in healthy Ross 308 chickens supplemented with 200 mg/kg peppermint for 42 days, with no significant change in the H/L ratio [98]. Other studies on newborn chickens and laying hens found no significant effect on leukocyte concentrations from peppermint supplements [72,75]. 

In addition to affecting leukocytes, menthol-containing supplements also influence antibody concentrations. Plasma IgG concentrations reduced after two weeks of 5% peppermint supplementation in Holstein steers [54] and after 56 days of 200 mg/kg peppermint supplementation in Habcock laying hens [72]. An increase in serum IgG and IgA was observed for laying hens supplemented with *Mentha arvensis*/*Geranium thunbergii* for 16 weeks [79]. Bai et al. observed increased serum IgG but not IgM in peppermint-supplemented laying hens [73]. Another study on newborn chickens reported a significantly reduced secondary antibody response to sheep red blood cells (SRBCs) after 400 mg/kg peppermint supplementation. The study also found evidence of duodenum cell destruction in supplemented animals [69]. The destructive effect at higher peppermint doses may allocate energy usage away from antibody production and into cell repair. Additionally, duodenum cell destruction can impair nutrient absorption, further depriving the host animal of nutrients essential for physiological functions such as immune response. This further supports the need to optimize menthol supplementation, as its antimicrobial effects may adversely affect the animal at high doses. However, some studies found no difference in serum antibody concentrations for young and adult chickens after supplementation [75,98]. Mehri et al. reported a linear increase in the humoral immune response against SRBCs and Newcastle disease virus in Japanese quail supplemented with peppermint at 10 k-40 k mg per kg of feed over a 5 week trial [81]. Further research is needed to understand menthol’s effect on livestock antibody production.

### 4.3. Anti-Oxidation

Reactive oxygen species (ROS) act as chemical messengers in inflammatory signaling and assist neutrophils in pathogen destruction [99,100,101]. ROS are also formed passively as by-products of oxidative processes in the mitochondria [102]. However, excess ROS are toxic to the host cells, causing DNA damage and lipid peroxidation that leads to cell death [103]. Menthol has been demonstrated to possess antioxidant properties, scavenging excess ROS through its hydroxyl groups [8,12,32]. Multiple studies in chickens [63,73] and cows [48,51] reported a significant elevation in serum antioxidant capacity in animals supplemented with peppermint and other menthol-containing compounds. Additionally, increased activity of ROS-scavenging enzymes, including catalase, glutathione peroxidase, and superoxide dismutase, was detected in supplemented livestock [48,73].

As previously discussed, Mehri et al. observed a decrease in MDA in the carcasses of young Japanese quail supplemented with various concentrations of peppermint, highlighting the antioxidant capacity of menthol-containing substances [81]. Similarly, in young male chickens supplemented with 2% spearmint, thiobarbituric acid reactive substances (TBARS) and 2,2-diphenyl-1-picrylhydrazyl (DPPH) levels were reduced [70]. Notably, these effects were not detected 21 days after birth but were observed at 42 days, potentially due to increased growth rate and oxidative stress during the latter period. In contrast, no significant differences in serum MDA were found in laying hens fed up to 0.4% peppermint for 28 days, though treated groups did possess lower average MDA concentrations than the control group [73]. In ruminants, Kholif et al. observed a reduction in serum MDA in lactating cattle fed 3–6 g Aromix^®^/day in a 3-month study [48], while they did not find differences in serum MDA, catalase, glutathione peroxidase, or superoxide dismutase in another trial with a similar experimental design [51]. 

Overall, studies suggest that menthol can reduce chronic inflammation and oxidative stress by regulating cytokine, leukocyte, antibody, and ROS concentrations (Table 2). More research is needed to optimize the menthol dosage and its combination with other plant extracts for different animal species to enhance its efficiency and consistency in enhancing the immunity of livestock.

## 5. Antimicrobial and Pesticidal Action of Menthol

Synthetic antibiotics have seen widespread usage in livestock farming worldwide to protect against illness and improve growth parameters. However, growing concerns have led to the banning or limiting of antibiotic use [18,104]. These concerns have arisen from two main factors. First, the overuse of antibiotics can lead to a buildup of toxic residues in the environment and in humans [104,105]. Second, excessive use of antibiotics promotes the development and propagation of antimicrobial-resistant (AMR) pathogens. AMR pathogens can be transmitted directly from livestock to humans through animal waste and food products. They can also transfer antimicrobial-resistance genes to otherwise susceptible environmental bacteria through horizontal gene transfer [19,104]. Preventing AMR is becoming of increasing importance, with human deaths from AMR pathogens estimated to be 10 million deaths per year by 2050 [106]. The antimicrobial properties of menthol and peppermint oil have been extensively studied in vitro [6,107,108]. These properties are primarily attributed to menthol’s ability to disrupt the lipid membrane of microorganisms, causing cellular leakage and subsequent death [31]. Because of this, menthol and other similar phytogenic compounds are garnering increased attention as potential alternatives to synthetic antibiotics, especially for use in livestock production (Table 3).

### 5.1. Antimicrobial Activity Against Skin Mucosal, Topical, and Environmental Pathogens

Several studies have found evidence for menthol’s efficacy against pathogens causing ruminant mastitis. In vitro antimicrobial activity of peppermint against *Escherichia coli* (*E. coli*) and *Staphylococcus aureus* (*S. aureus*) isolated from camel mastitis was observed, with minimum inhibitory concentrations (MIC) of 1.56% and 3.12% peppermint against *E. coli* and *S. aureus*, respectively [115]. Similarly, antimicrobial activity was observed against *Prototheca zopfii* (*P. zopfii*), a mastitis-causing alga isolated from cattle milk [116]. The alga was found to be susceptible to 50 µL of peppermint essential oil in vitro [117]. In contrast, a similar in vitro study did not identify any inhibitory activity of peppermint against *P. zopfii* at concentrations up to 30 µL/mL [118]. Another study found that peppermint exhibited limited bactericidal activity against *S. aureus* isolates from cow mastitis, though moderate inhibitory effects were observed against seven *S. aureus* strains and five *Staphylococcus chromogenes* strains [119]. In vivo, a decrease in *Pseudomonas aeruginosa* (*P. aeruginosa*) and *Candida albicans* (*C. albicans*) in the nasal mucosa of lactating cattle treated for 7 days with a phytogenic blend containing menthol and other compounds, such as cineol and chavicol (33.27% menthol mass fraction), was observed [111]. A tendency toward a reduction in the presence of *E. coli*, *P. aeruginosa*, *C. albicans*, and *S. aureus* in the vaginal mucosa of cattle treated with a similar blend (22.16% menthol mass fraction) was also detected. Additionally, the presence of several bacterial species, such as *Streptococcus uberis*, *P. aeruginosa*, *S. aureus*, and *C. albicans*, on cow teats was decreased after daily flushing with a blend including *M. piperita* and other phytogenic compounds [112]. In vivo results suggest that menthol-containing compounds hold significant potential as topical antibacterials, though effectiveness against pathogenic algae like *P. zopfii* remains questionable. One 42 day trial showed mesophilic bacteria, particularly Staphylococci, were significantly reduced in the environment after misting broiler houses with peppermint oil, suggesting peppermint could be effective as an aerosolized antimicrobial agent [113].

### 5.2. Bactericidal Activity Against Gut Pathogens

Gut pathogens can also be inhibited through feed supplementation. In a 12-week study, widespread changes in gastrointestinal bacteria populations were found in laying hens supplemented with Digestarom^®^, including a reduced abundance of several important pathogenic genera, such as Campylobacter, Staphylococcus, Fusobacterium, Desulfovibrio, Slackia, and Saccaropolyspora, while there was an increased abundance of *Porphyromonas*, *gingivalis*, and Gallibacterium [60]. Reduced *E. coli* and Campylobacter abundance was reported after Hubbard chickens were fed 3 kg of a phytogenic blend including peppermint per ton of feed after 42 days [61]. In Japanese quail, 10–40 peppermint/kg of feed reduced ileal coliform counts after 35 days [114]. 

While menthol compounds have shown great antimicrobial effectiveness in livestock, two studies call into question the efficacy of these products as substitutes for traditional antibiotics [109,110]. Aperce et al. reported a minimal reduction in total coliforms after cattle were supplemented with 0.3% menthol, but concerningly, an increase in tetracycline-resistant *E. coli* was observed after 4 weeks [109]. Similarly, Murray et al. found a tendency for increased tetracycline resistance in *E. coli* isolated from cattle supplemented with a blend of 0.3% menthol and 300 ppm of zinc [110]. However, there was no increase in AMR genes in chicken gut bacteria after 12 weeks of Digestarom^®^ supplementation [60]. Further studies are needed to investigate whether menthol specifically induces any AMR genes. 

### 5.3. Pesticidal Activity

There is evidence suggesting that menthol also possesses pesticidal activity. In vitro, larvicidal activity of peppermint was observed 3 days after spraying larva feed with peppermint oil. Fly density showed a 96% decrease around cattle topically treated with a 34.91 µg/µL peppermint formulation [120]. Similarly, 5% peppermint greatly repelled flies around cattle for up to 4 h after application [121]. *M. piperita* was found to be effective against water-buffalo-targeting lice, with a LC_50_ concentration of 12.35% [122], and against ticks with a LC_50_ concentration between 0.39 and 2.85% depending on life stage [123].

In summary, menthol products effectively control surface, mucosal, and gut pathogens, and peppermint shows efficacy against common cattle pests. However, further studies on pure menthol are needed to clarify individual potencies.

## 6. Impact of Menthol on Digestive Tract Microbial Ecosystem and Function

### 6.1. Impact on Rumen Microbial Ecosystem and Function

#### 6.1.1. Digestibility in Ruminants

Digestibility in ruminant systems can be tracked by direct methods such as fecal screening or the use of undigestible biomarkers like acid-insoluble ash [124,125]. Alternatively, measuring the total volume of gases produced during in vitro rumen studies serves as an effective indicator of digestive activity [124,126]. Gas production was not affected by the supplementation of up to 0.3% pure crystalline menthol in rumen fluid after 24 h of in vitro culture [127]. Other studies utilizing peppermint in the feed of ruminants, such as cattle, sheep, and buffalo, similarly reported no difference in indicators of digestive performance [52,57,58,128,129]. Despite menthol’s antimicrobial activity, potential improvements in ruminant digestion after menthol supplementation were also reported. Holstein steers that consumed 200 g of peppermint per day for 14 days possessed a tendency for increased nutrient digestibility [130]. Corresponding with their observations on feed intake, a pair of studies by Kholif et al. found improvements in the digestibility of various nutrients after lactating Friesian cattle were supplemented with 3 g/day of Aromix^®^ [48,51]. However, when supplementation increased to 6 g/day, digestibility decreased. Another study reported similar findings in vitro, as rumen fluid gas production increased after 1.5 µL/mL of peppermint supplementation but decreased at 3.0 µL/mL [131]. After 24 h in vitro, digestion in buffalo rumen fluid decreased after application of 600 ppm of peppermint; however, no change in digestion was observed at 30 or 300 ppm [132]. The findings of several studies suggest that menthol-containing supplements could impart beneficial effects on digestion at low doses but become detrimental at higher doses. Other researchers reported a decrease in ruminant digestion after peppermint concentrations ranging from 0.1 to 1.2 g/L of rumen fluid in vitro [133,134] and 5% of feed weight in vivo [53]. The decrease in digestion reported by several studies aligns with the observed decreases in commensal microbe populations.

#### 6.1.2. Modulation of Rumen Microbiota

The antimicrobial effects of menthol and other phenolic compounds do not selectively inhibit only pathogens but commensal microbes as well. Studies have observed wide-ranging shifts in rumen function from phytogenic compounds such as *M. piperita*, largely due in part to microbial modifications [135,136]. Alterations in the abundances of most rumen microbial taxa in growing sheep supplemented with OAX17 for 4 weeks were observed [137]. In vitro, peppermint supplementation ranging from 0.33 to 2 µL/mL and from 0.1 to 1 g/L decreased the relative abundance of prominent carbohydrate-degrading species in rumen fluid, including *Ruminococcus flavefaciens*, *Fibrobacter succinogenes*, *Prevotella ruminicola*, and *Ruminococcus albus* [133,138,139]. Overall decreases in bacteria, protozoa, methanogen, and archaea abundances were observed in the rumen in response to varying peppermint concentrations ranging from 0.1 to 1 g/L of digesta in vitro and 200 g/day in vivo [130,133,139]. In contrast, an increase in bacteria and methanogen abundance in vitro at 0.33 µL of peppermint per mL of buffalo rumen fluid was detected, but there was a decrease when peppermint was increased to 1–2 µL/mL [138]. As ruminants heavily rely on rumen microbes for degrading plant material, modifications in their abundances could affect nutrient digestion [140]. However, inhibiting methanogens holds the potential to reduce methane production and combat climate alterations [20,141].

#### 6.1.3. Ruminal pH Modulation

Menthol has the capacity to modulate the pH of the rumen. One of the significant concerns in ruminant nutrition is the development of sub-acute ruminal acidosis (SARA). Cattle can be fed diets high in starch but low in fiber to meet demanding energy requirements in conditions such as the lactating stage in dairy cattle and the fatting stage in beef cattle. However, this diet can often lead to a buildup of excess volatile fatty acids in the rumen and lead to lowered rumen pH (5.2–6.0) [142]. Persistent SARA can lead to a variety of conditions, including decreased feed intake, decreased milk production, poor body condition, diarrhea, and laminitis [142,143]. Aside from reducing the starch content of cattle diets, there is a desire for more productive methods to decrease SARA incidence. Amongst these, the use of essential oils as potential preventatives has garnered attention [144,145]. Nonlactating Holstein cattle under SARA conditions showed higher ruminal pH after 4 h of ad libitum feeding supplemented with 6.7 mg of menthol per kg of feed compared to those fed with a basal diet without menthol supplementation (pH 6.3 vs. pH 6.09), which was attributed to the reduction of volatile fatty acid production, particularly propionate [46]. Their findings on pH are supported by other in vivo and in vitro studies that supplemented ruminants with peppermint and other phytogenic blends [48,49,88,133,134].

Several of the aforementioned studies also found a decrease in propionate, supporting Castillo-Lopez et al.’s explanation for pH modification in cattle [49,88,133] and sheep [134]. However, a 3 month study supplementing lactating cattle with Aromix^®^ observed an increase in ruminal propionate alongside an increase in rumen pH from 6.71 in the control group compared to 6.82–6.85 in supplemented groups [48]. Other studies found no change in ruminal pH after phytogenic supplementation, with or without modified volatile fatty acid profiles [53,54,57,58,128,131,137], and another study found no short-term change to in vitro rumen pH supplemented with pure menthol at up to 0.3% of feed [127]. This suggests that factors other than volatile fatty acid production may also contribute to pH modulation. For example, a buffering effect from peppermint may explain rumen pH stabilization [52]. However, another study found a significant decrease in rumen pH from 14 days of peppermint supplementation at 200 g/day in dairy cattle, with peppermint-fed cows averaging 5.91 pH compared to 6.30 without supplement [130]. Consistent explanations for menthol’s pH-modulating abilities remain elusive. As such, while menthol-containing compounds have demonstrated the potential to attenuate rumen pH, more research must be carried out to better understand conflicting results and the mechanisms behind them.

#### 6.1.4. Methane Production and Acetate to Propionate (a/p) Ratio

Because of their antimicrobial activity, essential oils such as peppermint have garnered attention as potential controllers of methane emissions [20,146]. Several previously discussed studies that reported decreased digestibility after supplementation observed a corresponding decrease in methane production [53,131,133,134,138]. Other researchers observed a decrease in methane production without detrimental effects on digestibility. For instance, male buffalo fed a mixture of garlic bulb and peppermint (25 g of garlic bulb per 1 mL of peppermint oil) at 2.5% in feed over a 21-day period showed a 7% decrease in methane production [58]. Similarly, buffalo methane production decreased in vitro over 24 h without significantly affecting digestibility after up to 300 ppm of peppermint was added [132]. In contrast, a 4-week study observed no difference in rumen fermentation or methane production in unsupplemented sheep compared to sheep fed up to 160 mg/day of OAX17 [137]. A quadratic decrease in gas production but no decrease in methane production was observed when 100–400 mg of peppermint/L of rumen fluid collected from nonlactating cattle was supplemented in vitro [129]. These disparate results were attributed to differences in menthol concentration between individual peppermint plants, suggesting less efficiency in methane reduction when using mixed or impure menthol compounds such as peppermint oil.

Another proposed way of estimating digestive efficiency and methane production is based on the acetate to propionate (a/p) ratio. As carbohydrates are degraded by microbes, volatile fatty acids such as acetate, propionate, and butyrate are produced. As the ratio of acetate to propionate decreases, methane production generally decreases [147,148]. Several of the previously discussed studies observed an increase in the a/p ratio after supplementing ruminants with commercial phytogenic blends [49,88] or with peppermint [129,131,133,134,138]. However, as previously discussed, no studies reported an increase in methane production after supplementation, and many reported a decrease, conflicting with previous associations between a/p ratio and methane production. This was suggested to be due to an accumulation of molecular hydrogen due to peppermint reducing the hydrogen utilization efficiency for producing both volatile fatty acids and methane [134,138]. It is also possible that the general decrease in digestive function offsets increased methane production associated with a high a/p ratio. A decreased acetate-to-propionate (a/p) ratio was observed in cattle fed 3 g/day of Aromix^®^ compared to the control group, whereas an increased a/p ratio was reported at a dosage of 6 g/day [48,51]. As with several previous parameters, their studies suggest that menthol’s effects on biochemical parameters shift depending on dosage. Regardless, the a/p ratio is likely not a reliable marker for methane production in the context of supplementation with peppermint and other menthol-containing compounds.

#### 6.1.5. Ammonia Production

Another potential benefit of menthol’s antimicrobial activity is in modulating the ammonia concentration in the rumen. Proteolytic bacteria aid in the deamination and breakdown of proteins in ruminant guts. Ammonia is produced as a natural by-product of this process. However, when protein deamination produces more ammonia than the host can utilize, the excess ammonia is converted to urea and excreted, resulting in a loss of nitrogen sources. [139]. Excess urea excretion contributes to nitrogen-based pollution, including eutrophication and acid rain, when it is converted to reactive nitrogen compounds in the environment [149]. Many studies observed decreased ammonia concentrations, both in vivo and in vitro. Kholif et al. found a decrease in rumen ammonia after cattle were fed 3–6 g Aromix^®^/day [48,51]. Similarly, Holstein steers fed 200 g/day of peppermint for 14 days exhibited reduced rumen ammonia levels, which the researchers attributed to menthol’s antimicrobial activity [130]. Other cow and sheep studies using *M. piperita* support their findings [57,134]. In cattle supplemented with 0.04% of a menthol-containing blend, ammonia production initially increased compared to the control group after 2 weeks but rapidly declined by the third week [88]. However, Patra et al. reported no changes in ammonia production in sheep supplemented with up to 160 mg/day of OAX17 for 4 weeks [137]. Other ruminant studies utilizing peppermint similarly found no significant changes in ammonia production [52,128,129,132,133,139]. Only one study reported an increase in ruminal ammonia after 5% peppermint supplementation in Holstein steers [54]. 

Overall, menthol and related supplements have been repeatedly demonstrated to have a significant effect on rumen function. Primarily through their antimicrobial action, evidence suggests that feed digestibility, methane production, ammonia production, and rumen pH are all affected by the consumption of these compounds. However, differing methodologies leave optimal dosages of menthol unclear. Supplementation should be optimized to reduce unwanted by-products of digestion such as methane while minimizing negative effects on digestive performance.

### 6.2. Impact on Gut Microbial Ecosystem and Function in Poultry

#### 6.2.1. Digestibility in Poultry

In poultry, there is evidence suggesting that menthol could impart beneficial effects on digestion. Linear improvements to digestion in young Cobb chickens with increasing levels of supplementation with a menthol, anethol, and eugenol blend, where up to 150 mg of supplement per kg of feed increased dry matter digestibility, was identified [63]. This was attributed to microbial modulatory effects by Mentha species. Similar improvements to crude protein digestibility were seen after peppermint supplementation to chickens at 200 mg/kg of feed and up to 296 mg/kg, respectively [69,77], which was linked to increases in bile secretion [150]. However, two other studies found no improvements to digestibility, apart from cysteine [62], after chickens were supplemented up to 2% of feed with *Mentha cordifolia* and 150 mg of a menthol/anethol blend per kg of feed [62,70]. Studies on poultry digestion are limited, but the lack of detrimental findings suggests further study on menthol-containing compounds could yield beneficial findings. A single study by [70] also observed improvements in poultry ammonia excretion, where spearmint feeding at 0.5–2% of feed weight for 42 days significantly reduced ammonia excretion in chickens.

#### 6.2.2. Modulation of Gut Microbiota in Poultry

Studies on the effects of menthol on gut microbiota in poultry are relatively limited, though some research has directly reported modifications in specific gut microbial populations. After young Hubbard chickens were supplemented with 3 kg of a mint-containing blend per ton of feed for 42 days, abundances of commensal Lactobacillus and Enterococcus bacteria in the ileum and Enterococcus in the cecum were increased compared to the control group [61]. In another study, decreased abundances of commensal genera such as Lactobacillus and Ruminococcus were reported after the administration of Digestarom^®^ to laying hens [60]. However, the concentration of menthol in this product is unclear. While tracking menthol and peppermint’s effects on microbe populations could serve as a helpful predictor of digestive performance, the ultimate value of their modifications is indicated by the resulting effects on digestive function.

Overall, menthol-containing supplements appear to impart beneficial effects on poultry digestion, especially for protein (Table 4). However, other aspects of digestion are largely uninvestigated.

## 7. Impact of Menthol on Metabolism

Given the alterations to digestion by menthol and peppermint found by many studies, supplementation also leads to changes in the overall metabolic profiles of animals. These include alterations in serum parameters, such as urea, cholesterol, total protein, and others. (Table 5).

### 7.1. Serum Urea

The presence of urea in blood serum is inextricably linked to ammonia. Ammonia is either produced by protein breakdown in the body or absorbed through the gastrointestinal tract [154]. A buildup of ammonia is toxic to the body, so the urea cycle in the liver converts ammonia to urea, which is then excreted in urine [155]. As previously discussed, several studies reported decreased ammonia in the rumens of livestock after feed supplementation. Thus, it is possible that serum urea levels could be affected in turn. Some results support this notion in ruminants. When Kholif et al. supplemented cattle with 3 or 6 g/day of Aromix^®^ for 3 months, they observed a significant decrease in serum urea (20.3–21.4 g/dL) at both doses compared to the control group (22.8 g/dL), which they attributed to reduced protein catabolism [48]. Similarly, sheep supplemented with 3% dried peppermint over a 90-day period had lower serum urea concentrations throughout the trial, reaching significance during the middle period, which was attributed to a reduction in rumen ammonia [57]. In another study, lactating Holstein–Friesian cattle supplemented with 1.2 g/day of BTX12 had reduced serum urea and urinary ammonia levels, also likely due to antimicrobial activity against proteolytic bacteria [50]. It is speculated that a TRP channel-stimulated increase in protonated ammonium cation absorption over ammonia could reduce blood pH, stimulating glutamine synthesis through liver glutamine synthetase. An increase in glutamine, as a key form of ammonia transport for excretion and amino acid synthesis, could facilitate protein synthesis for lactating cattle [50]. This explanation aligns with other observed effects of menthol, such as increased serum protein levels and TRP-facilitated ion absorption. In contrast, another study found that serum urea levels increased after 5% peppermint supplementation to Holstein steers over 2 weeks, aligning with an observed increase in rumen ammonia [54]. While differing in results, the study affirms the link between levels of rumen ammonia and serum urea. Other studies found no difference in serum urea concentration in cattle and sheep [51,56]; however, despite not reporting a significant difference in serum urea, Patra et al. reported an increase in serum amino acids such as glutamine, glutamate, and aspartate in supplemented Suffolk sheep. They also attributed this to ammonium absorption upregulating glutamine synthetase, lending further evidence to menthol’s potential to improve protein efficiency.

### 7.2. Serum Protein and Amino Acids

Coinciding with serum urea reduction, there is evidence suggesting serum protein concentration is increased by menthol-containing compound supplementation. As discussed previously, Patra et al. observed an increase in serum amino acids after supplementing sheep with OAX17 [56]. In agreement with their findings on blood urea, Kholif et al. also found an increase in blood protein levels compared to controls for lactating cattle fed 3–6 g/day of Aromix^®^ [48]. In a related study, serum protein levels after 3 g/day of Aromix^®^ treatment were increased, even with no change to serum urea concentration [51]. However, other ruminant studies found no effect on serum protein after peppermint feeding [54,89], even with reduced blood urea [50,57]. These discrepancies could be due to factors such as animal breed, supplement differences, and other physiological variables [89].

Poultry studies similarly show evidence of the regulation of blood protein concentration by menthol. Chickens supplemented with peppermint ranging from 74 to 296 mg per kg of feed showed a linear increase in serum protein levels [77]. Other studies similarly reported increased serum protein in chickens after peppermint supplementation [65,73,76]. These results reflect improvements in poultry feed digestibility, specifically for protein, as discussed previously. However, like ruminants, several studies found no changes in serum protein, suggesting other factors also play a role [61,63,72,75,98,152].

### 7.3. Serum Glucose

Menthol impacts on another important blood metabolite, glucose, were also investigated in several studies. Consistent with their findings on feed intake and nutrient digestibility, Kholif et al. reported dose-dependent effects of Aromix^®^ on cattle serum glucose levels, where cattle fed 3 g daily had higher serum glucose levels than the control group, and an increase to 6 g/day led to a decrease in serum glucoses [48,51]. Specifically, they attribute the increased level of serum glucose to an increase in precursor molecules such as propionate after 3 g of supplementation, suggesting menthol’s effect on serum glucose is mediated through the regulation of carbohydrate digestion in the rumen. Another study observed an increase in serum glucose from 54.57 mg/dL in control animals to 70.73 mg/dL in rams fed 30 mL of peppermint oil per kg of feed [89]. However, increased serum glucose or rumen volatile fatty acid concentrations were not found in another study supplementing cattle with peppermint at 5% of diet [54]. Similarly, Patra et al. found no difference in the serum glucose levels of sheep supplemented with up to 160 mg of OAX17 per day [56]. However, they found that sheep fed menthol-containing compounds before slaughter displayed a tendency for improved glucose uptake in the absence of Na^+^. Thus, they suggest menthol may have stimulatory effects on Na^+^ independent glucose transporters (GLUT), such as GLUT-2 and GLUT-5. Given menthol’s action on TRP ion channels, stimulatory effects on other transporters may be possible [156]. Unlike protein concentrations, poultry studies generally found no difference in serum glucose levels after supplementation [63,65,72,77,78,98]. Overall, evidence suggests menthol compounds may significantly increase blood glucose levels through various mechanisms in ruminants, but evidence suggests the same may not be the case in poultry.

### 7.4. Serum Triglycerides and Cholesterol

Like humans, livestock are susceptible to lipid-related diseases. These diseases are often associated with serum levels of cholesterol and triglycerides. Dairy cattle are particularly susceptible to fatty liver disease, where fat is directed to the liver in response to lactation energy deficits. Decreased serum cholesterol and triglyceride levels have been associated with fatty liver disease in cattle [157]. In poultry, decreased serum lipid profiles are considered preferable for the quality and health of the meat [158,159].

Studies suggest menthol-containing compounds can impact cholesterol and related biomolecules in ruminants. Kholif et al.’s studies on cattle found significant decreases in serum cholesterol levels, as well as triglycerides and total lipids, after Aromix^®^ supplementation [48,51]. 3-hydroxy-3-methylglutaryl-CoA reductase (HMGCR) catalyzes the reaction converting HMG-CoA to mevalonate, a key precursor to cholesterol [160]. As a key irreversible reaction in cholesterol synthesis, the reaction catalyzed by HMGCR is the primary site of cholesterol regulation. Menthol and other monoterpenes have demonstrated the ability to suppress the activity and synthesis of HMGCR [161,162,163,164]. While Kholif et al. treated cattle with the blended supplement Aromix^®^, they concluded that the phytogenic compounds in the supplement must impart most of the cholesterol-suppressing effect, based on previous studies [48]. Some other ruminant studies did not find significant changes in the serum levels of cholesterol or related lipoproteins after supplementation with peppermint [54,89] or OAX17 [56], suggesting that factors other than menthol are likely to contribute to the regulation of these molecules as well.

However, there exists an even greater body of evidence for menthol’s cholesterol-modulating activity in poultry studies. Serum cholesterol level was lower in quail supplemented with 10–40 g of peppermint supplementation per kg of feed compared with control birds (2.92–3.47 nmol/L vs. 3.85–3.00 nmol/L) [81]. A decrease in low-density lipoprotein (LDL) and triglycerides and an increase in high-density lipoprotein (HDL) were also observed in supplemented quail [81]. Reduced serum cholesterol and other lipid metabolites after peppermint and menthol-containing blended supplements were also observed in numerous chicken trials at various life stages [65,76,77,98]. One study reported significantly decreased cholesterol, triglyceride, and lipoprotein profiles in quail fed 1–4% spearmint or *Mentha spicata* (*M. spicata*) for 28 days [153]. While the study also acknowledged the possible role of HMGCR suppression in these effects, it also reported an increase in Lactobacillus species in the guts of quail supplemented with the plant. Prior research associated Lactobacillus presence in the gut with an improved cholesterol profile [165]. *M. spicata* possesses relatively little menthol compared to *M. piperita*, but a different study achieved similar results for chickens supplemented with 3 kg of a peppermint-containing phytogenic blend per ton of feed for 42 days, supporting menthol’s role in the observed effects of *M. spicata* [61]. The study reported decreased blood cholesterol and increased Lactobacillus presence in the small intestine of treated animals compared to controls. Thus, it is likely that menthol’s effects on cholesterol also stem from its gut bacteria modulation activity. Other chicken studies observed a decrease in cholesterol and triglyceride levels when peppermint supplementation was paired with thyme and chromium [78,80]. A few poultry studies did not find a decrease in cholesterol and lipid levels from peppermint and other menthol-containing supplements [63,72,75]. In contrast to most studies, Bai et al. reported that 28 days of 0.1–0.4% peppermint supplementation in laying hens tended to increase serum cholesterol and significantly increased triglycerides compared to control hens [73]. The reason for this increase is not entirely clear. Despite this, there is a significant body of evidence suggesting that menthol can serve as an effective mediator of serum cholesterol and lipid profiles in ruminants and poultry.

### 7.5. Serum Calcium

Finally, menthol has been shown to improve calcium absorption and serum calcium levels in ruminants. Ruminants, especially dairy cattle, often experience a condition known as hypocalcemia, or milk fever. Hypocalcemia occurs from a disruption in calcium homeostasis, causing a deficiency in blood calcium levels [166]. A lack of available calcium can cause a range of physiological dysfunctions, especially with muscle function. Hypocalcemia often occurs around calving and lactation periods due to the high calcium demands of milk production [167]. Menthol’s agonistic activity on TRP channels, such as the cold-sensing TRPM8 channel, has been extensively demonstrated [8]. TRP channel signaling is carried out through cations such as Ca^2+^ and has been found to play a significant role in calcium absorption and homeostasis [168,169]. As such, menthol has been investigated as a potential preventer of hypocalcemia. A pair of in vitro studies demonstrated pure menthol’s ability to stimulate calcium absorbance in cow and sheep rumen epithelia [170,171]. It was found that 10–100 µmol/L applied to epithelial cells was able to increase the absorption of Ca^2+^ ions. Through overexpressing the ion channel bTRPV3, intracellular Ca^2+^ levels rose in response to menthol stimulation, likely by binding to and stabilizing the channel’s open state [171]. Another study affirmed these findings, finding a prominent presence of TRPV3 in the rumen of sheep [172]. They also demonstrated enhanced transcellular Ca^2+^ transport through rumen epithelial cells treated with 50 µM menthol. Additionally, rumen epithelial cells from sheep fed 80–160 mg of OAX17 per day before slaughter showed an enhanced response to menthol stimulation in vitro, with greater tissue conductivity. The study suggests that extended exposure to menthol can prime these TRP channels for greater future cation absorption. Significant Ca^2+^ absorption and TRPV3 channels were not detected in the jejunum, suggesting that menthol’s calcium stimulatory effects occur primarily in the rumen, as opposed to the intestines [172].

In vivo studies found a rise in serum calcium, coinciding with Ca^2+^ absorption observed in vitro. Serum calcium levels were significantly higher in cattle fed 1.2 g/day of BTX12 over two 20-day periods (2.53 nmol/L) compared to the control group (2.46 nmol/L), but urine calcium levels did not differ between the groups, indicating enhanced calcium absorption without substantially increased excretion [50]. Other studies of cattle supplemented with 400 g/day of peppermint [151] and 3–6/day of Aromix^®^ [51] found a significant increase in serum calcium levels, suggesting this effect can be produced with a wide range of menthol-containing supplements. A 28-day trial reported a tendency for serum calcium to increase in sheep fed 160 g/day of OAX17, but not in sheep fed only 80 g/day [56]. Furthermore, increased calcium digestibility was observed in the rumens of sheep fed 3% peppermint [57]. The results of these studies suggest that menthol can stimulate calcium absorption not only through direct interaction with TRP channels but also through the modulation of pH and nutrient digestibility. However, a different study by Kholif et al. did not observe effects on serum calcium [48], and poultry studies did not observe significant alterations in serum calcium profiles after peppermint supplementation [65,72,77].

Overall, several serum parameters can be altered by the consumption of menthol-containing compounds. Calcium and various lipids are chief among them, with urea, protein, and glucose also potentially being regulated.

## 8. Current Gaps and Future Directions

Despite current studies on menthol’s effects in livestock, many future avenues for study and elaboration still exist. There is a particularly notable gap in the current literature investigating the use of menthol for its cooling and pain-relieving properties in livestock. As previously discussed, one of menthol’s most established properties is its ability to stimulate TRPM8 cold receptors [8,173]. When used in conjunction with other cooling strategies such as fans and misters, menthol holds the potential to further offset animal heat stress. Some of the previous studies utilized phytogenic supplementation in animals under heat stress conditions, where subsequent improvements in behavior, immunity, nutrition, and serum were observed [49,71,76,80]. However, the quantity of literature investigating the effects of menthol and heat stress together is limited, and menthol’s effects on stress biomarkers such as cortisol remain unreported. There is evidence for heat stress in cattle resulting in increased cortisol, adrenaline, and various pro-inflammatory cytokines [86,174]. Furthermore, human and animal trials suggest that menthol can mitigate the physiological effects of heat stress, including elevated cortisol [175,176,177]. As such, topical or oral administration of menthol holds the potential to improve livestock welfare in hot climates. Furthermore, menthol’s analgesic properties [37] may possess utility in painful procedures such as cattle dehorning and castration.

Another limitation of previous studies arises from the use of Mentha products and mixed phytogenic blends over pure menthol. While peppermint’s primary active ingredient is menthol, the plant also contains prominent compounds such as menthone and menthyl acetate, as well as numerous others such as acetaldehyde, pinene, etc. [178,179]. Additionally, menthol concentration varies, even between individual plants of the same species, with one study reporting concentrations ranging from 35.01 to 47.50% [180]. Furthermore, the percentage of menthol correlates with the plant’s antibacterial effectiveness [107,119]. While most studies utilizing these “impure” supplements obtained results comparable to those using pure menthol, their results may be unreliable in the context of elucidating the effects of menthol alone.

There are other yet-unknown factors that may contribute to menthol’s effects on livestock, such as understudied metabolites of menthol, especially oxidized products, and their potential impacts on animals. As previously discussed, menthol is primarily absorbed in the small intestine [29]. Differences in digestive system physiology and gastrointestinal microbiomes between ruminants and monogastric animals may influence their responses to menthol supplementation. For instance, while menthol’s antimicrobial properties are well established, it remains largely unexplored whether microbes in the rumen or hindgut can degrade menthol into specific metabolites, which may then be absorbed through the rumen, small intestine, or large intestine and subsequently regulate host physiology.

Finally, optimal dosages for menthol supplementation in livestock production remain unclear. Given menthol’s antimicrobial properties, overuse has the potential to detrimentally affect host digestion and metabolic equilibrium. Higher doses of menthol can also cause damage to the eukaryotic cells of the animal. Varying results were reported between studies for most physiological and metabolic parameters. Differences in menthol supplementation dosages and experimental protocols likely contributed to differing results between studies. The varied menthol forms, from pure menthol to peppermint and commercial supplement blends, also confound the search for an ideal dosage. Thus, more standardized supplementation procedures would be ideal for future studies seeking to optimize the dosage of these compounds for different animals.

## 9. Conclusions

In conclusion, menthol holds promise for modulating behavioral, microbial, and biochemical parameters in ruminant and poultry livestock, just as it has in humans. Physiologically and economically important effects include alterations to feed intake and feeding behavior, improved FCR and animal product quality, increased absorption of ions such as Ca^2+^ and ammonium, decreased pro-inflammatory cytokines and ROS, destruction of harmful arthropods and microorganisms on and within the animal, reduced methane production, and alterations to feed digestibility and various serum nutrient profiles. Beyond these effects, there remain other underexplored applications for livestock, such as managing heat stress and pain response. Further research into optimizing this supplement for use in a wider livestock industry setting could help alleviate growing issues in livestock production, such as heat stress, antimicrobial resistance, and nitrogen and methane pollution.

## Figures and Tables

**Table 1:** Impact of menthol and menthol-rich supplements on feed intake, growth performance, and animal production.

Animal	Growth Stage/Condition ^1^	Sample Size ^2^	Treatment	Dosage ^3^	Experiment Duration	Effects of Treatment ^4^	Reference
Holstein Cattle	nonlactating, subacute ruminal acidosis conditions	4 CON, 4 per TRT (4 × 4 incomplete Latin square)	Mint or Menthol	T1: 15.3 mg Mnt/kg T2: 153 mg Mnt/kg T3: 6.7 mg Mthl/kg T4: 67 mg Mthl/kg	4 h of supplement	Tendency towards a linear decrease in feed intake from mint, tendency towards a linear increase in feeding time from menthol.	[46]
Holstein Cattle	nonlactating	4 CON, 4 per TRT (4 × 4 incomplete Latin square)	Mint or Menthol	T1: 15.3 mg Mnt/kg T2: 153 mg Mnt/kg T3: 6.7 mg Mthl/kg T4: 67 mg Mthl/kg	4 h of supplement	Increased salivation rate.	[47]
Friesian Cattle	lactating	10 CON, 10 per TRT	Aromix^®^ (133 mg menthol/g)	T1: 3 g/day T2: 6 g/day	3 months	Increased feed intake, milk yield, and milk contents with T1; decrease in same parameters with T2.	[48]
Holstein Cattle	lactating, heat stress conditions	16 CON, 16 TRT	BioHerbal^®^ (contains peppermint)	2 g/day	28 days	Increased feed intake, increased milk yield.	[49]
Holstein–Friesian Cattle	lactating	36 CON, 36 TRT	BTX12 (>80% menthol)	1.2 g/day	Two periods of 20 days	No effect on feed intake, increased milk yield.	[50]
Friesian Cattle	lactating	10 CON, 10 per TRT	Aromix^®^ (133 mg menthol/g)	T1: 3 g/day T2: 6 g/day	3 months	No effect on feed intake, increased milk yield with T1.	[51]
Holstein Cattle	lactating	4 CON, 4 TRT	Peppermint	5%	22 days	Increased feeding time, no effect on feed intake, no effect on milk yield or quality.	[52,53]
Holstein Cattle	NA	4 CON, 4 TRT (4 × 4 Latin square)	Peppermint	5%	2 weeks	Increased feed intake.	[54]
Suffolk Sheep	growing	8 CON, 8 per TRT	OAX17 (90% menthol)	T1: 80 mg/day T2: 160 mg/day	28 days	Increased feed intake and feeding time, no effect on body weight.	[55,56]
Sanjabi Sheep	90 days old	6 CON, 6 TRT	Peppermint	3%	90 days	Increased feed intake, no effect on FCR.	[57]
Murrah Buffalo	NA	2 CON, 2 TRT (2 × 2 switch over)	Garlic Bulb + Peppermint	2.5%	Two periods of 21 days	No effect on feed intake.	[58]
Ross 308 Chickens	1 day old	96 CON, 48 per TRT	Peppermint or Menthol	T1: 5 g Pep/kg T2: 10 g Pep/kg T3: 15 g Pep/kg T4: 26 mg Mthl/kg T5: 52 mg Mthl/kg T6: 78 mg Mthl/kg	Two periods of 35 days	Increased feed intake and body weight, improved FCR, decreased abdominal fat.	[59]
Chickens	18 week old laying hens	20K CON, 20K TRT	Digestarom^®^ (contains menthol)	150 g/t	12 weeks	Decreased mortality and dirty eggs.	[60]
Hubbard Chickens	1 day old	50 CON, 50 per TRT	Phytogenic Blend (garlic, mint, etc.) or Blend + Organic Acids	T1: 3 kg phytogenic blend/ton T2: 3 kg phytogenic blend/ton + organic acids	42 days	Increased feed intake, increased body weight, improved FCR.	[61]
Cobb 500 Chickens	1 day old males	Trial 1: 160 CON, 160 TRT; Trial 2: 40 CON, 40 TRT	Phytogenic Blend (menthol, anethole)	150 mg/kg	Trial 1: 42 days Trial 2: 21 days	No effect on feed intake, body weight, or FCR.	[62]
Cobb Chickens	1 day old	75 CON, 75 per TRT	Phytogenic Blend (menthol, anethol, and eugenol)	T1: 100 mg/kg T2: 150 mg/kg	42 days	No effect on feed intake, improved FCR.	[63]
Ross 308 Chickens	1 day old	60 CON, 60 per TRT	Phytogenic Blend (ziziphora, oregano, peppermint)	T1: 1% ziziphora, 0.5% others T2: 1% oregano, 0.5% others T3: 1% Pep, 0.5% others	42 days	Decreased feed intake, no change in body weight, improved FCR, and no change to carcass traits with T3. Other groups saw improvements in parameters.	[64]
Hy-Line Brown Chickens	64 week old laying hens	50 CON, 50 per TRT	Peppermint	T1: 5 mg/kg T2: 10 mg/kg T3: 15 mg/kg T4: 20 mg/kg	12 weeks	Increased feed intake, improved FCR, increased egg rate, weight, and Haugh score.	[65]
Hubbard Chickens	1 day old	40 CON, 40 per TRT	Peppermint	T1: 0.25% T2: 0.5% T3: 1% T4: 1.5%	6 weeks	Increased feed intake, increased body weight, improved FCR.	[66]
Chickens	1 week old	104 CON, 104 TRT	Peppermint	0.2% (~70 mg/kg Mthl)	42 days	No effect on feed intake, increased abdominal fat.	[67]
Ross 308 Chickens	240 days old	48 CON, 48 per TRT	Peppermint	T1: 0.1% T2: 0.2% T3: 0.3% (in water)	42 days	No effect on feed intake, improved FCR, increased carcass yield, decreased abdominal fat.	[68]
Ross 308 Chickens	1 day old	48 CON, 48 per TRT	Peppermint	T1: 200 mg/kg T2: 400 mg/kg	6 weeks	No effect on feed intake, improved FCR with T1.	[69]
Chickens	1 day old	9 CON, 9 per TRT	Spearmint	T1: 0.5% T2: 1% T3: 1.5% T4: 2%	42 days	No change in final feed intake, body weight, or FCR, increased production index, decreased abdominal fat.	[70]
Chickens	1 day old, heat stress conditions	48 CON, 48 per TRT	Peppermint	T1: 1% T2: 2%	42 days	No effect on feed intake, no effect on FCR, reduced heat stress.	[71]
Habcock Chickens	21 week old laying hens	36 CON, 36 per TRT	Peppermint	T1: 50 mg/kg T2: 100 mg/kg T3: 200 mg/kg T4: 50 mg/L T5: 100 mg/L T6: 200 mg/L	56 days	No effect on feed intake, improved FCR, no effect on egg quantity/quality, affected yolk color at 28 days.	[72]
Chickens	60 week old laying hens	54 CON, 54 per TRT	Peppermint	T1: 0.1% T2: 0.2% T3: 0.3% T4: 0.4%	28 days	No effect on feed intake, increased eggshell thickness and egg saturated fatty acids.	[73]
Ross 308 Chickens	1 day old	30 CON, 30 per TRT	Peppermint	T1: 5 g/kg T2: 10 g/kg T3: 15 g/kg	35 days	Decreased feed intake and body weight with T3. No effect on FCR or carcass characteristics.	[74]
Ross 308 Chickens	1 day old	48 CON, 48 per TRT	Peppermint	T1: 4 g/kg T2: 8 g/kg	42 days	Increased body weight and improved FCR with T1, no effect on carcass.	[75]
Ross 308 Chickens	1 day old, heat stress conditions	80 CON, 80 per TRT	Peppermint	T1: 200 ppm ethanolic/kg body weight T2: 200 ppm nano-/kg body weight T3: 200 ppm micro-/kg body weight	42 days	Improved FCR, no effect on feed intake or body weight.	[76]
Bovans Brown Chickens	32 week old laying hens	40 CON, 40 per TRT	Peppermint	T1: 74 mg/kg T2: 148 mg/kg T3: 222 mg/kg T4: 296 mg/kg	12 weeks	Improved FCR, increased egg production, weight, shell thickness, and Haugh score.	[77]
Lohmann LSL-Lite Chickens	40 week old laying hens, cold stress conditions	30 CON, 30 per TRT	Peppermint or Peppermint + Thyme	T1: 100 mg/kg diet Pep T2: 100 mg/kg diet Pep + thyme	8 weeks	Increased feed intake, improved FCR from combined, increased shell thickness from combined.	[78]
ISA Brown Chickens	28 week old laying hens	24 CON, 24 per TRT	Wild Mint + Geranium	T1: 0.01% T2: 0.05% T3: 0.1%	16 weeks	Increased feed intake, increased egg production, weight, and Haugh score.	[79]
Cobb 500 Chickens	1 day old, heat stress conditions	60 CON, 60 per TRT	Peppermint or Peppermint + Chromium Picolinate	T1: 250 mg Pep/kg T2: 250 mg Pep/kg + chromium picolinate	42 days	No effect on feed intake or body weight, improved FCR with T2.	[80]
Japanese Quail	8 days old	120 CON, 60 per TRT	Peppermint	T1: 10 g/kg T2: 20 g/kg T3: 30 g/kg T4: 40 g/kg	5 weeks	Decreased MDA, cooking loss, and drip loss.	[81]

^1^ NA: Not available/not applicable. ^2^ CON: Control group, TRT: Treatment group. ^3^ T: Treatment group number, Mthl: Menthol, Pep: Peppermint, Mnt: Mint. ^4^ FCR: Feed conversion ratio, MDA: Malondialdehyde.

**Table 2:** Anti-inflammation, immune system modulation, and anti-oxidation properties of menthol.

Animal	Growth Stage/Condition ^1^	Sample Size ^2^	Treatment	Dosage ^3^	Experiment Duration ^4^	Effects of Treatment ^5^	Reference
Friesian Cattle	lactating	10 CON, 10 per TRT	Aromix^®^ (133 mg menthol/g)	T1: 3 g/day T2: 6 g/day	3 months	Increased total antioxidant capacity, catalase, superoxide dismutase, and glutathione peroxidase, decreased MDA.	[48]
Friesian Cattle	lactating	10 CON, 10 per TRT	Aromix^®^ (133 mg menthol/g)	T1: 3 g/day T2: 6 g/day	3 months	Increased total antioxidant capacity, no effect on catalase, superoxide dismutase, glutathione peroxidase, or MDA.	[51]
Holstein Cattle	nonlactating	4–5 CON, 4–5 TRT	Digestarom^®^ (contains menthol)	0.04%	Two periods of 6 weeks	Reduced serum amyloid A in week 3.	[88]
Holstein Cattle	NA	4 CON, 4 TRT (4 × 4 Latin square)	Peppermint	5%	2 weeks	Decreased IgG. No significant effect on total antioxidant capacity.	[54]
Suffolk Sheep	growing	8 CON, 8 per TRT	OAX17 (90% menthol)	T1: 80 mg/day T2: 160 mg/day	28 days	Decreased neutrophils. No effect on leukocytes, lymphocytes, monocytes, erythrocytes, or platelets.	[56]
Sheep	3 to 4 years-old	4 CON, 4 per TRT	Peppermint	T1: 10 mL/kg T2: 20 mL/kg T3: 30 mL/kg	NA	Increased white blood cells at higher doses.	[89]
Cobb Chickens	1 day old	75 CON, 75 per TRT	Phytogenic Blend (menthol, anethol, and eugenol)	T1: 100 mg/kg T2: 150 mg/kg	42 days	Decreased IL-18 in the spleen. No effect on spleen TGF-β4, IFN-γ, IL-10, IL-2, or iNOS. No effect on tonsil cytokines. Tendency to increase total antioxidant capacity.	[63]
Hubbard Chickens	1 day old	40 CON, 40 per TRT	Peppermint	T1: 0.25% T2: 0.5% T3: 1% T4: 1.5%	6 weeks	Decreased H/L ratio. No effect on total white blood cells.	[66]
Ross 308 Chickens	1 day old	48 CON, 48 per TRT	Peppermint	T1: 200 mg/kg T2: 400 mg/kg	6 weeks	Decreased secondary antibody response with T2.	[69]
Chickens	1 day old	9 CON, 9 per TRT	Spearmint	T1: 0.5% T2: 1% T3: 1.5% T4: 2%	42 days	Decreased 2,2-diphenyl-1-picrylhydrazyl and thiobarbituric acid at 42 days.	[70]
Habcock Chickens	21 week old laying hens	36 CON, 36 per TRT	Peppermint	T1: 50 mg/kg T2: 100 mg/kg T3: 200 mg/kg T4: 50 mg/L T5: 100 mg/L T6: 200 mg/L	56 days	Decreased IgG with T6. No effect on lymphocytes, neutrophils, monocytes, or platelets.	[72]
Chickens	60 week old laying hens	54 CON, 54 per TRT	Peppermint	T1: 0.1% T2: 0.2% T3: 0.3% T4: 0.4%	28 days	Increased total antioxidant capacity, IgG, and superoxide dismutase. No effect on MDA or IgM.	[73]
Ross 308 Chickens	1 day old	48 CON, 48 per TRT	Peppermint	T1: 4 g/kg T2: 8 g/kg	42 days	No effect on white blood cells, heterophils, lymphocytes, or antibodies.	[75]
Ross 308 Chickens	1 day old, heat stress conditions	80 CON, 80 per TRT	Peppermint	T1: 200 ppm ethanolic/kg body weight T2: 200 ppm nano-/kg body weight T3: 200 ppm micro-/kg body weight	42 days	Decreased H/L ratio.	[76]
ISA Brown Chickens	28 week old laying hens	24 CON, 24 per TRT	Wild Mint + Geranium	T1: 0.01% T2: 0.05% T3: 0.1%	16 weeks	Increased serum IgA, IgG, IL-6, and TNF-α.	[79]
Ross 308 Chickens	chicks	75 CON, 75 per TRT	Peppermint or Peppermint + Artichoke	T1: 200 mg Pep/kg water T2: 200 mg Pep/kg water + 1.5% artichoke	42 days	Increased heterophils and lymphocytes. No change to H/L ratio or antibodies.	[98]
Japanese Quail	8 days old	120 CON, 60 per TRT	Peppermint	T1: 10 g/kg T2: 20 g/kg T3: 30 g/kg T4: 40 g/kg	5 weeks	Linear increase in humoral immune response, decreased MDA.	[81]

^1^ NA: Not available/not applicable, LPS: Lipopolysaccharide. ^2^ CON: Control group, TRT: Treatment group. ^3^ T: Treatment group number, Pep: Peppermint. ^4^ NA: Not available/not applicable, LPS: Lipopolysaccharide, Mthl: Menthol. ^5^ LPS: Lipopolysaccharide, MDA: Malondialdehyde, H/L: Heterophil/lymphocyte.

**Table 3:** Antimicrobial and pesticidal action of menthol.

Animal	Growth Stage/Condition ^1^	Sample Size ^2^	Treatment	Dosage ^3^	Experiment Duration	Effects of Treatment ^4^	Reference
Holstein Cattle	NA	13 CON, 13 TRT	Menthol	0.3%	4 weeks	Increased tetracycline-resistant *E. coli*. No effect on total coliform counts or other antimicrobial resistances.	[109]
Cattle	NA	20 CON, 20 per TRT	Menthol or Menthol + Supra-Nutritional Zinc	T1: 0.3% Mthl T2: 0.3% Mthl + 300 ppm zinc	3 weeks	Tendency for increased tetracycline-resistant *E. coli* and MDR with T2, increased macrolide-resistant *Enterococci* in first 21 days from menthol.	[110]
Cattle	lactating	20 per TRT	Phytogenic Blends (menthol, chavicol, cineol, etc.)	T1: 10 mL/d nasal T2: 20 mL/d vaginal	7 days	Decreased nasal *P. aeruginosa* and *C. albicans*. Reduced vaginal *E. coli*, *P. aeruginosa*, *C. albicans*, and *S. aureus*.	[111]
Cattle	cattle with signs of udder lesions	14 TRT	Phytogenic Blend (*S. sclarea*, *M. canadensis*, *M. piperita* and *C. sativum*)	5–6 mL/nipple/day	7 days	Decreased nipple *Str. uberis*, *P. aeruginosa*, *S. aureus*, *E. coli*, *H. somni*, and *C. albicans*.	[112]
Chickens	18 week old laying hens	50 CON 50 TRT	Digestarom^®^ (contains menthol)	150 g/t	12 weeks	Decreased gut *Campylobacter*, *Staphylococcus*, *Fusobacterium*, *Desulfovibrio*, *Slackia*, *Saccaropolyspora*, etc. Increased *P. gingivalis* and *Gallibacterium.* No effect on antimicrobial resistance.	[60]
Hubbard Chickens	1 day old	50 CON 50 per TRT	Phytogenic Blend (garlic, mint, etc.) or Blend + Organic Acids	T1: 3 kg phytogenic blend/ton T2: 3 kg phytogenic blend/ton + organic acids	42 days	Decreased gut *E. coli* and *Camoylobacter*.	[61]
Ross 308 Chickens	1 day old	120 CON 120 TRT	Peppermint	Room fogged with 1 L of 1:250 or 1:500 oil aerosol	42 days	Decreased environmental mesophiles, and *Staphylococci*.	[113]
Japanese Quail	8 days old	72 CON 72 per TRT	Peppermint	T1: 10 g/kg T2: 20 g/kg T3: 30 g/kg T4: 40 g/kg	35 days	Reduced ileal coliform counts.	[114]

^1^ NA: Not available/not applicable. ^2^ CON: Control group, TRT: Treatment group, NA: Not available/not applicable. ^3^ T: Treatment group number, Mthl: Menthol. ^4^ MDR: Multidrug resistance, MIC: Minimum inhibitory concentration, LC_50_: Lethal concentration 50.

**Table 4:** Impact of menthol on digestive tract microbial ecosystem and function.

Animal	Growth Stage/Condition ^1^	Sample Size ^2^	Treatment	Dosage ^3^	Experiment Duration	Effects of Treatment ^4^	Reference
Holstein Cattle	nonlactating, subacute ruminal acidosis conditions	4 CON, 4 per TRT (4 × 4 incomplete Latin square)	Mint or Menthol	T1: 15.3 mg Mnt/kg T2: 153 mg Mnt/kg T3: 6.7 mg Mthl/kg T4: 67 mg Mthl/kg	4 h of supplement	Increased mean rumen pH from menthol treatment with high-concentrate SARA diet.	[46]
Holstein Cattle	rumen fluid from 17 month olds tested in vitro	5 CON, 5 per TRT	Menthol	T1: 0.003% T2: 0.03% T3: 0.3%	24 h	No effect on gas production, rumen pH, or VFAs.	[127]
Friesian Cattle	lactating	10 CON, 10 per TRT	Aromix^®^ (133 mg menthol/g)	T1: 3 g/day T2: 6 g/day	3 months	Increased nutrient digestibility and VFAs with T1, decreased nutrient digestibility and increased propionate with T2, decreased ammonia production and increased pH at both doses.	[48]
Holstein Cattle	lactating, heat stress conditions	16 CON, 16 TRT	BioHerbal^®^ (contains peppermint)	2 g/day	28 days	Decreased propionate, increased a/p ratio and rumen pH, no effect on total VFAs.	[49]
Friesian Cattle	lactating	10 CON, 10 per TRT	Aromix^®^ (133 mg menthol/g)	T1: 3 g/day T2: 6 g/day	3 months	Increased nutrient digestibility and decreased a/p ratio with T1, decreased nutrient digestibility and increased a/p ratio with T2, decreased ammonia production at both doses.	[51]
Holstein Cattle	nonlactating	4–5 CON, 4–5 TRT	Digestarom^®^ (contains menthol)	0.04%	Two periods of 6 weeks	Increased rumen pH, decreased propionate and increased a/p ratio and other VFAs at 2 weeks, increased ammonia production at 2 weeks, decreased ammonia production at 3 weeks.	[88]
Holstein Cattle	lactating	4 CON, 4 TRT	Peppermint	5%	22 days	No effect on nutrient digestibility, VFAs, or gas production, immediate decrease in ammonia production but not after 3 h, minor decrease in rumen pH.	[52]
Holstein Cattle	NA	4 CON, 4 TRT (4 × 4 Latin square)	Peppermint	5%	2 weeks	Increased ammonia production, no effect on rumen pH or VFAs.	[54]
Holstein Cattle	lactating	4 CON, 4 TRT	Peppermint	5%	22 days	Decreased nutrient digestibility and methane production per dry/digestible matter intake, no effect on VFAs or pH.	[53]
Cattle	nonlactating cow rumen fluid tested in vitro	3 CON, 3 per TRT	Peppermint	T1: 100 mg/L T2: 200 mg/L T3: 300 mg/L T4: 400 mg/L	48 h	Tendency for decreased digestibility, increased a/p ratio, no effect on methane production or ammonia production.	[129]
Holstein Cattle	NA	4 CON, 4 TRT	Peppermint	200 g/day	14 days	Tendency for increased nutrient digestibility, decreased ammonia production, VFAs, rumen protozoa, and rumen pH.	[130]
Jersey Cattle	rumen fluid tested in vitro from fistulated lactating cattle	3 CON, 3 per TRT	Peppermint	T1: 0.1 g/L T2: 0.25 g/L T3: 1 g/L	24 h	Decreased archaea, protozoa, methanogens, cellulolytic bacteria, gas production, methane production, and propionate, increased a/p ratio and rumen pH, no change to ammonia production.	[133]
Jersey Cattle	rumen fluid tested in vitro from fistulated lactating cattle	3 CON, 3 TRT	Peppermint	1 g/L	24 h	Decrease in most bacteria and archaea, increase in *S. bovis*, no effect on ammonia production.	[139]
Suffolk Sheep	growing	8 CON, 8 per TRT	OAX17 (90% menthol)	T1:80 mg/day T2: 160 mg/day	4 weeks	Alterations in ruminal abundance of most bacterial taxa, no effect on rumen fermentation, pH, VFAs, ammonia, or methane.	[137]
Sanjabi Sheep	90 days old	6 CON, 6 TRT	Peppermint	3%	90 days	No effect on nutrient digestibility or pH, decreased ammonia production.	[57]
Buffalo	NA	3 CON, 3 TRT (3 × 3 Latin square)	Peppermint	2 mL/100 kg body weight	24 days	No effect on nutrient digestibility, pH, VFAs, ammonia production, or rumen microbes.	[128]
Buffalo	rumen fluid tested in vitro	5 CON, 5 per TRT	Peppermint	T1: 1.5 µL/mL T2: 3 µL/mL	24 h	Increased gas production with T1, decreased gas production, methane production, dry matter digestibility, and increased a/p ratio with T2, no effect on pH.	[131]
Buffalo	rumen fluid tested in vitro	NA	Peppermint	T1: 30 ppm T2: 300 ppm T3: 600 ppm	24 h	Increased propionate with T3, decreased digestibility and VFAs with T3, decreased methane production with T2 and T3, no effect on ammonia production.	[132]
Sheep	rumen fluid tested in vitro	3 CON, 3 per TRT	Peppermint	T1: 100 mg/L T2: 200 mg/L T3: 400 mg/L T4: 800 mg/L T5: 1200 mg/L	96 h	Decreased organic matter digestibility, methane production, ammonia production, and propionate, increased a/p ratio and pH.	[134]
Buffalo	rumen fluid tested in vitro	6 CON, 6 per TRT	Peppermint	T1: 0.33 µL/mL T2: 1 µL/mL T3: 2 µL/mL	24 h	Decreased bacteria, fungi, and methanogen populations with T2 and T3, increased with T1, increased a/p ratio, decreased feed digestibility and methane production.	[138]
Murrah Buffalo	NA	2 CON, 2 TRT (2 × 2 switch over)	Garlic Bulb + Peppermint	2.5% of dry weight	Two periods of 21 days	Decreased methane production, no effect on dry matter digestibility, rumen pH, or rumen microbes.	[58]
Chickens	18 week old laying hens	50 CON, 50 TRT	Digestarom^®^ (contains menthol)	150 g/t	12 weeks	Decreased *Lactobacillus* and *Ruminococcus*.	[60]
Hubbard Chickens	1 day old	50 CON, 50 per TRT	Phytogenic Blend (garlic, mint, etc.) or Blend + Organic Acids	T1: 3 kg phytogenic blend/ton T2: 3 kg phytogenic blend/ton + organic acids	42 days	Increased gut *Lactobacillus* in ileum and *Enterococcus* in cecum and ileum.	[61]
Cobb 500 Chickens	1 day old	Trial 1: 160 CON, 160 TRT; Trial 2: 40 CON, 40 TRT	Phytogenic Blend (menthol, anethole)	150 mg/kg diet	Trial 1: 42 days Trial 2: 21 days	Increased cysteine digestibility, no effect on other nutrient digestibility.	[62]
Cobb Chickens	1 day old	75 CON, 75 per TRT	Phytogenic Blend (menthol, anethol, and eugenol)	T1: 100 mg/kg T2: 150 mg/kg	42 days	Linear increase in dry matter digestibility and apparent metabolizable energy corrected for nitrogen, no effect on crude protein or ether extract digestibility.	[63]
Ross 308 Chickens	1 day old	48 CON, 48 per TRT	Peppermint	T1: 200 mg/kg T2: 400 mg/kg	6 weeks	Increased crude protein digestibility with T1, no effect on dry matter or ether extract digestibility.	[69]
Chickens	1 day old	9 CON, 9 per TRT	Peppermint	T1: 0.5% T2: 1% T3: 1.5% T4: 2%	42 days	Decreased ammonia excretion, no effect on nutrient digestibility.	[70]
Bovans Brown Chickens	32 week old laying hens	40 CON, 40 per TRT	Peppermint	T1: 74 mg/kg T2: 148 mg/kg T3: 222 mg/kg T4: 296 mg/kg	12 weeks	Linear increase in crude protein and ether extract digestibility, no effect on other nutrient digestibility.	[77]

^1^ NA: Not available/not applicable. ^2^ NA: Not available/not applicable, CON: Control group, TRT: Treatment group. ^3^ T: Treatment group number, Mthl: Menthol, Mnt: Mint. ^4^ SARA: Subacute ruminal acidosis, VFA: Volatile fatty acid, a/p = Acetate/propionate.

**Table 5:** Impact of menthol on serum parameters.

Animal	Growth Stage/Conditions ^1^	Sample Size ^2^	Treatment	Dosage ^3^	Experiment Duration ^4^	Effects of Treatment ^5^	Reference
Friesian Cattle	lactating	10 CON, 10 per TRT	Aromix^®^ (133 mg menthol/g)	T1: 3 g/day T2: 6 g/day	3 months	Decreased serum urea, triglycerides, cholesterol, and total lipids, increased serum protein, increased serum glucose with T1, decreased glucose with T2, no effect on serum calcium.	[48]
Holstein–Friesian Cattle	lactating	36 CON, 36 TRT	BTX12 (>80% menthol)	1.2 g/day	Two periods of 20 days	Decreased serum and urine urea, increased serum calcium, no effect on serum protein or urine calcium.	[50]
Friesian Cattle	lactating	10 CON, 10 per TRT	Aromix^®^ (133 mg menthol/g)	T1: 3 g/day T2: 6 g/day	3 months	Increased serum protein and glucose with T1, decreased serum glucose with T2, decreased serum cholesterol and triglycerides, increased serum calcium, no effect on serum urea.	[51]
Holstein Cattle	NA	4 CON, 4 TRT (4 × 4 Latin square)	Peppermint	5%	2 weeks	Increased serum urea, no effect on serum protein or glucose, cholesterol, triglycerides, or lipoproteins.	[54]
Holstein Cattle	4 year olds, transition state pregnancy	10 CON, 10 TRT	Peppermint	400 g/day	15 days + 12 h after calving	Increased serum calcium and decreased urine pH at end of 15 days and 12 h after calving.	[151]
Suffolk Sheep	growing	8 CON, 8 per TRT	OAX17 GmbH (90% menthol)	T1: 80 mg/day T2: 160 mg/day	28 days	Tendency for increased serum calcium with T2, increased serum glutamine, glutamate, asparagine, and aspartate, no effect on serum urea, glucose, cholesterol, or triglycerides, tendency for improved Na^+^ independent glucose uptake.	[56]
Sanjabi Sheep	90 days old	6 CON, 6 TRT	Peppermint	3%	90 days	Decreased serum urea and increased serum glucose and triglycerides during middle period, no effect on serum protein or cholesterol, increased calcium digestibility.	[57]
Sheep	3 to 4 years-old	4 CON 4 per TRT	Peppermint	T1: 10 mL/kg T2: 20 mL/kg T3: 30 mL/kg	NA	Increased serum glucose with T3, no effect on serum protein, cholesterol, or triglycerides.	[89]
Hubbard Chickens	1 day old	50 CON, 50 per TRT	Phytogenic Blend (garlic, mint, etc.) or Blend + Organic Acids	T1: 3 kg phytogenic blend/ton T2: 3 kg phytogenic blend/ton + organic acids	42 days	Decreased serum cholesterol, no effect on serum protein.	[61]
Cobb Chickens	1 day old	75 CON, 75 per TRT	Phytogenic Blend (menthol, anethol, and eugenol)	T1: 100 mg/kg T2: 150 mg/kg	42 days	No effect on serum protein, glucose, or cholesterol.	[63]
Hy-Line Brown Chickens	64 week old laying hens	50 CON, 50 per TRT	Peppermint	T1: 5 mg/kg T2: 10 mg/kg T3: 15 mg/kg T4: 20 mg/kg	12 weeks	Increased serum protein, decreased serum cholesterol, no effect on serum glucose.	[65]
Habcock Chickens	21 week old laying hens	36 CON, 36 per TRT	Peppermint	T1: 50 mg/kg T2: 100 mg/kg T3: 200 mg/kg T4: 50 mg/L T5: 100 mg/L T6: 200 mg/L	56 days	No effect on serum protein, glucose, cholesterol, lipoproteins, or calcium.	[72]
Chickens	60 week old laying hens	54 CON, 54 per TRT	Peppermint	T1: 0.1% T2: 0.2% T3: 0.3% T4: 0.4%	28 days	Increased serum protein, uric acid, and triglycerides, trend towards increased serum cholesterol, no effect on serum lipoproteins or urea.	[73]
Ross 308 Chickens	1 day old	48 CON, 48 per TRT	Peppermint	T1: 4 g/kg T2: 8 g/kg	42 days	No effect on serum protein, cholesterol, or lipoproteins.	[75]
Ross 308 Chickens	1 day old, heat stress conditions	80 CON, 80 per TRT	Peppermint	T1: 200 ppm ethanolic/kg body weight T2: 200 ppm nano-/kg body weight T3: 200 ppm micro-/kg body weight	42 days	Increased serum protein and HDL, decreased serum cholesterol, triglycerides, LDL, and VLDL.	[76]
Bovans Brown Chickens	32 week old laying hens	40 CON, 40 per TRT	Peppermint	T1: 74 mg/kg T2: 148 mg/kg T3: 222 mg/kg T4: 296 mg/kg	12 weeks	Increased serum protein, decreased serum cholesterol, no effect on serum glucose or calcium.	[77]
Lohmann LSL-Lite Chickens	40 week old laying hens, cold stress conditions	30 CON, 30 per TRT	Peppermint or Peppermint + Thyme	T1: 100 mg Pep/kg diet T2: 100 mg Pep/kg + thyme	8 weeks	No effect on serum glucose or uric acid decreased serum triglycerides with T2.	[78]
Cobb 500 Chickens	1 day old females, heat stress conditions	60 CON, 60 per TRT	Peppermint or Peppermint + Chromium Picolinate	T1: 250 mg Pep/kg T2: 250 mg Pep/kg + chromium picolinate	42 days	Decreased serum glucose and triglycerides with T2, no effect on serum cholesterol or lipoproteins.	[80]
Ross 308 Chickens	chicks	75 CON, 75 per TRT	Peppermint or Peppermint + Artichoke	T1: 200 mg Mnt/kg water T2: 200 mg Mnt/kg water + 1.5% artichoke	42 days	Decreased serum cholesterol and triglycerides, no effect on serum urea, protein, glucose, or lipoproteins.	[98]
Cobb Chickens	1 day old	20 CON, 20 per TRT	Peppermint or Peppermint + Black Seed	T1: 300 mg/kg Pep T2: 300 mg/kg Pep + 1 mL/kg black seed	42 days	No effect on serum urea or protein.	[152]
Japanese Quail	8 days old	120 CON, 60 per TRT	Peppermint	T1: 10 g/kg T2: 20 g/kg T3: 30 g/kg T4: 40 g/kg	5 weeks	Decreased serum cholesterol, triglycerides, and LDL, increased serum HDL.	[81]
Japanese Quail	7 days old	60 CON, 60 per TRT	Spearmint	T1: 1% T2: 2% T3: 3% T4: 4%	28 days	Decreased serum cholesterol, triglycerides, and LDL.	[153]

^1^ NA: Not available/not applicable. ^2^ NA: Not available/not applicable, CON: Control group, TRT: Treatment group. ^3^ T: Treatment group number, Pep: Peppermint, Mnt: Mint. ^4^ NA: Not available/not applicable. ^5^ HDL: High-density lipoprotein, LDL: Low-density lipoprotein, VLDL: Very low-density lipoprotein, TRP: Transient receptor potential.
